# Supplementary material for: Cellular traits regulate fluorescence-based light-response phenotypes of coral photosymbionts living in-hospite
Source: Front Physiol. 2023 Oct 11;14:1244060. doi: 10.3389/fphys.2023.1244060 (PMC10598705; doi:10.3389/fphys.2023.1244060)
Supplement: Supplementary file 1 [file DataSheet1.PDF]

Supplemental Information

Supplemental Table 1: Average C, N, and P content and ratios for symbionts across coral hosts. Each measurement is an average of 3 replicates, with +/- 1 standard deviation from the mean. Where a standard deviation is not present, fewer than 3 replicates were available.

	Symbiont	Host	C per Cell (pmol)	N per Cell (pmol)	P per Cell (pmol)	C:N	N:P	C:P
**Outdoor**	**C3**	*A.hum* var 1	136.37 +/- 20.72	20.57 +/- 4.99	1.26 +/- 0.31	6.71 +/- 0.56	16.49 +/- 0.81	110.59 +/- 8.85
	**C3**	*A.mill*	322.46 +/- 159.63	41.80 +/- 23.22	1.15 +/- 0.66	7.87 +/- 0.96	27.34 +/- 1.31	228.66 +/- 29.17
	**C21**	*Acropora* sp	151.57 +/- 96.62	21.13 +/- 13.84	0.90 +/- 0.05	7.29 +/- 0.47	22.58 +/- 14.30	161.77 +/- 98.5
	**C3**	*A.val*	369.08 +/- 233.10	39.30 +/- 27.29	1.24 +/- 0.83	9.68 +/- 0.86	27.37 +/- 2.44	263.71
	**C21**	*A.yong*	136.13 +/- 7.97	18.50 +/- 1.15	0.62 +/- 0.03	7.36 +/- 0.14	30.03 +/- 1.71	220.97 +/- 13.47
	**C1**	*C.chal*	202.31 +/- 20.06	22.20 +/- 1.30	1.04 +/- 0.26	9.10 +/- 0.51	21.73 +/- 4.76	199.39 +/- 53.09
	**C26**	*M.cap* var 1	300.37 +/- 45.76	32.55 +/- 6.01	1.75 +/- 0.22	9.27 +/- 0.32	19.04 +/- 7.63	175.23 +/- 64.59
	**C15**	*M.cap* var 2	135.37 +/- 41.99	21.27 +/- 6.93	0.64 +/- 0.17	6.40 +/- 0.13	33.10 +/- 4.21	211.35 +/- 22.91
	**C1**	*M.cap* var 3	193.62 +/- 14.30	23.35 +/- 1.77	0.88 +/- 0.20	8.30	29.87 +/- 0.24	247.76 +/- 1.91
	**C15**	*M.dig*	229.39 +/- 13.16	27.30 +/- 1.73	0.92	8.43 +/- 0.49	28.25 +/- 2.83	234.26 +/- 5.44
	**C1**	*P.cact*	154.91 +/- 29.47	22.33 +/- 7.16	0.98 +/- 0.24	7.14 +/- 1.02	22.6 +/- 2.39	159.97 +/- 8.26
	**D1**	*P.cont*	185.32 +/- 4.30	26.93 +/- 4.71	1.16 +/- 0.13	7.00 +/- 1.02	25.10 +/- 1.78	171.41 +/- 23.12
	**D1**	*P.dam* var 1	204.08 +/- 104.56	28.83 +/- 13.49	2.04	6.99 +/- 0.33	21.54	157.51
	**D1**	*P.dam* var 2	257.14 +/- 42.22	37.10 +/- 7.23	1.82 +/- 0.34	6.96 +/- 0.25	20.27 +/- 1.01	141.02 +/- 6.67
	**D1**	*T.ren* var 1	168.46	28.70	1.12	5.52 +/- 0.98	29.73 +/- 5.28	161.05 +/- 14.54
	**D1**	*T.ren* var 2	414.87 +/- 44.20	60.00 +/- 9.31	2.32 +/- 0.54	6.95 +/- 0.44	24.79 +/- 1.25	177.91 +/- 16.38
**Indoor**	**C3**	*A.hum* var 2	182.29 +/- 39.75	28.63 +/- 1.22	1.51 +/- 0.38	6.34 +/- 1.17	20.17 +/- 4.02	124.93 +/- 6.74
	**C15**	*M.cap* var 2	171.06 +/- 37.21	22.83 +/- 3.78	0.70 +/- 0.08	7.45 +/- 0.40	30.58 +/- 3.55	221.02 +/- 18.15
	**C15**	*M.cap* var 3	171.80 +/- 23.94	18.75 +/- 2.05	0.65 +/- 0.12	9.31 +/- 2.34	28.78 +/- 6.28	260.64 +/- 9.00
	**D1**	*T.ren* var 2	113.50 +/- 59.54	15.47 +/- 8.17	0.75 +/- 0.48	7.34 +/- 0.04	23.96 +/- 8.26	175.95 +/- 60.91

Supplemental Table 2: Average values for cellular metrics determined via flow cytometry for *Symbiodiniaceae* across coral hosts. Each measurement is an average of 3 replicates, with +/- 1 standard deviation from the mean. Where a standard deviation is not present, fewer than 3 replicates were available for that metric. Neutral lipid content is reported in Fluorescence Units (FU).

	Symbiont	Host	Neutral Lipids (FU)	Bead-Norm Chl *a*	Bead-Norm FSC (Cell Size)	Bead-Norm SSC (Granularity)
**Outdoor**	**C3**	*A.hum* var 1	1418.67 +/- 716.38	6.32 +/- 0.11	3.67 +/- 0.28	1.96 +/- 0.14
	**C3**	*A.mill*	477.33 +/- 826.77	20.39 +/- 0.17	3.32 +/- 0.17	2.55 +/- 0.09
	**C21**	*Acropora* sp	3015.00 +/- 1727.71	26.83 +/- 1.85	3.60 +/- 0.40	1.49 +/- 0.03
	**C3**	*A.val*	1894.00 +/- 934.05	21.35 +/- 1.26	3.61 +/- 0.16	3.08 +/- 0.41
	**C21**	*A.yong*	6606.00 +/- 7338.48	25.95 +/- 1.20	3.77 +/- 0.06	2.01 +/- 0.09
	**C1**	*C.chal*	6047.67 +/- 540.51	20.52 +/- 0.58	3.73 +/- 0.15	2.53 +/- 0.05
	**C26**	*M.cap* var 1	2461.67 +/- 1118.45	30.65 +/- 0.65	3.74 +/- 0.59	2.86 +/- 0.43
	**C15**	*M.cap* var 2	2352.33 +/- 668.13	87.33 +/- 110.69	3.78 +/- 0.10	3.16 +/- 0.39
	**C1**	*M.cap* var 3	1895.33 +/- 710.88	23.92 +/- 1.39	3.79 +/- 0.12	3.39 +/- 0.31
	**C15**	*M.dig*	1749.00 +/- 248.57	21.60 +/- 0.39	3.66 +/- 0.12	2.84 +/- 0.16
	**C1**	*P.cact*	2049.33 +/- 1776.15	20.11 +/- 0.55	3.89 +/- 0.19	2.02 +/- 0.11
	**D1**	*P.cont*	NA	21.47 +/- 0.14	4.52 +/- 0.42	2.91 +/- 0.30
	**D1**	*P.dam* var 1	7640.33 +/- 1245.23	18.19 +/- 0.85	4.22 +/- 0.59	2.25 +/- 0.67
	**D1**	*P.dam* var 2	7389.33 +/- 6651.45	22.10 +/- 2.02	4.68 +/- 0.80	2.42 +/- 0.30
	**D1**	*T.ren* var 1	10020.00	20.06	5.21	3.79
	**D1**	*T.ren* var 2	1652.00 +/- 2861.35	22.68 +/- 1.53	5.34 +/- 0.51	3.75 +/- 0.30
**Indoor**	**C3**	*A.hum* var 2	955.67 +/- 460.65	21.44 +/- 0.87	3.23 +/- 0.09	2.57 +/- 0.23
	**C15**	*M.cap* var 2	2248.33 +/- 1143.01	19.93 +/- 0.95	3.67 +/- 0.12	2.69 +/- 0.13
	**C15**	*M.cap* var 3	1485.00 +/- 81.07	22.41 +/- 0.27	3.49 +/- 0.07	3.68 +/- 0.03
	**D1**	*T.ren* var 2	7916.67	18.55 +/- 0.18	4.20 +/- 0.03	2.54 +/- 0.01

Supplemental Table 3: Bonferroni-adjusted p-values comparing photophysiology across excitation wavelength within each of the 4 phenotypic profiles. These statistics correspond with the phenotypic profiles found in Figure 3. Significant comparisons across excitation wavelengths appear in bold.

	λ (nm)	λ (nm)	Φ_{PSII}	σ_{PSII}	qP	NPQ	τ_1	τ_2
Phenotype 1	442	420	4.05E-01	**<2e-16**	1.000	**2.69E-12**	**<2e-16**	1
	458	420	**2.22E-15**	**<2e-16**	1.000	**2.00E-16**	**<2e-16**	**2.00E-16**
	505	420	1	**<2e-16**	1.000	**2.00E-16**	**<2e-16**	**2.00E-16**
	525	420	**2.22E-15**	**<2e-16**	1.000	**2.00E-16**	**<2e-16**	**2.00E-16**
	458	442	**2.00E-16**	**<2e-16**	1.000	**0.000126**	**<2e-16**	**2.00E-16**
	505	442	**0.00432**	**<2e-16**	1.000	**1.33E-11**	**<2e-16**	**2.00E-16**
	525	442	**2.00E-16**	**<2e-16**	1.000	**6.09E-05**	**<2e-16**	**2.00E-16**
	505	458	**1.97E-10**	**<2e-16**	1.000	6.45E-02	**<2e-16**	**4.60E-08**
	525	458	1	**<2e-16**	1.000	1	**<2e-16**	1
	525	505	**1.76E-10**	**<2e-16**	1.000	1.03E-01	1	**8.44E-11**
Phenotype 2	442	420	**5.60E-03**	**2.00E-16**	1.000	**1.45E-12**	**2.00E-16**	1
	458	420	1	**2.00E-16**	1.000	**2.00E-16**	**2.00E-16**	**2.00E-16**
	505	420	**1.24E-06**	**2.00E-16**	2.66E-01	**2.00E-16**	**2.00E-16**	**0.015262**
	525	420	1	**2.00E-16**	1.000	**0.007534**	**2.00E-16**	**2.00E-16**
	458	442	**1.33E-03**	**2.00E-16**	1.000	1	**2.00E-16**	**2.00E-16**
	505	442	0.6621	**2.00E-16**	1.000	**0.001224**	**2.00E-16**	**2.16E-05**
	525	442	**1.19E-02**	**2.00E-16**	1.000	**0.000576**	**2.00E-16**	**2.00E-16**
	505	458	**1.53E-07**	**4.64E-08**	1.000	0.056438	**0.00616**	**2.00E-16**
	525	458	1	**2.00E-16**	1.000	**3.47E-06**	**4.44E-15**	**0.000259**
	525	505	**3.80E-06**	**2.00E-16**	1.000	**3.77E-14**	**2.88E-05**	**2.00E-16**
Phenotype 3	442	420	**7.59E-06**	**2.00E-16**	1	**2.00E-16**	**2.88E-07**	**0.00121**
	458	420	**0.00698**	**2.00E-16**	1	**2.00E-16**	**2.00E-16**	1
	505	420	**2.00E-16**	**2.00E-16**	0.799	**2.00E-16**	**2.00E-16**	**1.73E-07**
	525	420	**1.21E-06**	**2.00E-16**	1	**2.00E-16**	**2.00E-16**	1
	458	442	1	**2.00E-16**	1	0.3355	**1.13E-13**	**0.00421**
	505	442	**0.00754**	**2.00E-16**	0.93	**4.34E-06**	**2.00E-16**	0.73067
	525	442	1	**2.00E-16**	1	1	**2.00E-16**	**0.00227**
	505	458	**8.46E-06**	**0.000476**	1	**0.0341**	**0.02048**	**1.04E-06**
	525	458	0.57103	**2.00E-16**	1	0.4726	**8.59E-10**	1
	525	505	**0.02512**	**2.00E-16**	1	**9.02E-06**	**0.00657**	**4.26E-07**
Phenotype 4	442	420	**2.90E-02**	**<2e-16**	1	**3.69E-04**	**2.00E-16**	1
	458	420	1	**<2e-16**	1	**5.78E-08**	**2.00E-16**	**<2e-16**
	505	420	**1.61E-07**	**<2e-16**	1.62E-01	**2.00E-16**	**2.00E-16**	1
	525	420	1	**<2e-16**	5.48E-01	**1.73E-05**	**2.00E-16**	**<2e-16**
	458	442	**3.32E-03**	**<2e-16**	1	0.897146	**2.00E-16**	**<2e-16**
	505	442	0.075415	**<2e-16**	1	**3.99E-09**	**2.00E-16**	5.02E-01
	525	442	1	**<2e-16**	1	1	**2.00E-16**	**<2e-16**
	505	458	**3.82E-09**	**2.09E-02**	1	**5.17E-05**	**1.83E-08**	**<2e-16**
	525	458	4.40E-01	**<2e-16**	1	1	**2.00E-16**	1
	525	505	**2.16E-04**	**<2e-16**	1	**2.16E-07**	1.40E-01	**<2e-16**

Supplemental Table 4: Bonferroni-adjusted p-values comparing photophysiology across phenotype for each excitation wavelength. These statistics correspond with the phenotypic profiles found in Figure 3. Significant comparisons appear in bold.

	Phen	Phen	Φ_{PSII}	σ_{PSII}	qP	NPQ	τ_1	τ_2
420 nm	2	1	**1.21E-09**	1	**1.30E-07**	**2.00E-16**	**1.13E-03**	**2.30E-10**
	3	1	1	1	1	**6.97E-06**	4.58E-01	1
	4	1	**6.05E-12**	1	**1.49E-05**	**3.59E-11**	5.53E-02	**7.43E-09**
	3	2	**2.93E-05**	0.353	**0.000226**	**2.33E-05**	1	**2.24E-04**
	4	2	1	1	1	**5.04E-07**	1	1
	4	3	**2.37E-06**	1	**0.005604**	1	1	**0.002287**
442 nm	2	1	**2.66E-15**	1	**1.99E-07**	**2.00E-16**	**3.87E-04**	**4.39E-12**
	3	1	**0.015948**	1	1	**3.70E-09**	**1.04E-02**	**2.15E-03**
	4	1	**2.00E-16**	1	**1.19E-05**	**6.38E-09**	1.32E-01	**8.86E-11**
	3	2	**0.001686**	1	**0.00302**	**0.00126**	1	0.13268
	4	2	1	1	1	**1.52E-10**	3.28E-01	1
	4	3	**0.000253**	1	**0.03625**	0.78251	0.956649	0.51449
458 nm	2	1	**2.00E-16**	1	**2.44E-08**	**2.00E-16**	1.41E-01	**1.55E-12**
	3	1	**4.38E-06**	1	1	**3.93E-07**	**1.28E-04**	**1.60E-06**
	4	1	**2.00E-16**	0.433	**1.02E-05**	**1.83E-05**	8.81E-02	**2.43E-12**
	3	2	0.0963	1	**8.46E-05**	**4.84E-03**	9.79E-02	1
	4	2	1	1	0.94526	**1.98E-10**	1	1
	4	3	**0.0435**	1	**0.00465**	0.38599	0.087283	1
505 nm	2	1	**2.00E-16**	1	**2.27E-08**	**2.00E-16**	**3.06E-03**	**3.21E-11**
	3	1	**2.54E-06**	1	1	**9.47E-07**	**3.16E-06**	**1.11E-06**
	4	1	**2.00E-16**	1	**1.45E-05**	**2.16E-05**	**2.03E-02**	**9.93E-12**
	3	2	0.3312	1	**2.21E-05**	**2.80E-02**	1.76E-01	1
	4	2	1	1	0.80667	**4.28E-08**	1	1
	4	3	**0.0407**	0.491	**0.00198**	0.521	**0.02679**	1
525 nm	2	1	**2.00E-16**	1	**1.47E-08**	**2.00E-16**	1.20E-01	**1.73E-14**
	3	1	**1.78E-07**	1	1	**2.43E-04**	**1.47E-05**	**3.05E-08**
	4	1	**2.00E-16**	1	**2.05E-05**	**1.22E-03**	**4.40E-02**	**1.33E-15**
	3	2	0.7822	1	**7.93E-06**	**2.82E-02**	**2.94E-02**	1
	4	2	1	1	0.60249	**9.95E-07**	1	1
	4	3	0.0979	0.115	**0.00135**	1	**0.0383**	1
